# Effective “Pop-Out” Nucleus Technique in Cataract Surgery With Pseudoexfoliation: A Case Report

**DOI:** 10.7759/cureus.111999

**Published:** 2026-07-03

**Authors:** Talal Sharaf

**Affiliations:** 1 Ophthalmology, Emirates Hospital, Dubai, ARE

**Keywords:** capsular tension ring, cataract surgery, complex cataract, hydrodissection, nucleus prolapse technique, phacoemulsification, pseudoexfoliation syndrome, zonular weakness

## Abstract

Pseudoexfoliation syndrome (PXF) presents significant challenges in cataract surgery due to zonular weakness and poor pupillary dilation. We report a case of a 72-year-old woman with PXF who underwent cataract surgery using a “pop-out” nucleus technique facilitated by hydrodissection. The technique allowed the safe prolapse of the nucleus into the anterior chamber despite zonular instability. Postoperative visual outcomes were favorable, improving from 0.1 preoperatively to 0.9 one week after surgery. This case highlights the safety and effectiveness of the “pop-out” technique in managing complex cataract cases associated with pseudoexfoliation.

## Introduction

Pseudoexfoliation syndrome (PXF) is a systemic condition characterized by the accumulation of fibrillar extracellular material in ocular tissues, particularly on the lens capsule and zonules [1,2]. It is commonly associated with poor pupillary dilation, zonular weakness, and an increased risk of complications during cataract surgery [3].

Managing cataract in eyes with pseudoexfoliation presents significant surgical challenges, including zonular instability and a higher risk of capsular rupture [2,4]. Various surgical techniques have been described to improve safety and outcomes in such cases.

We present a case demonstrating the use of a “pop-out” nucleus technique facilitated by hydrodissection in a patient with pseudoexfoliation syndrome, highlighting its effectiveness and safety in the presence of zonular weakness.

## Case presentation

**Figure 1:**
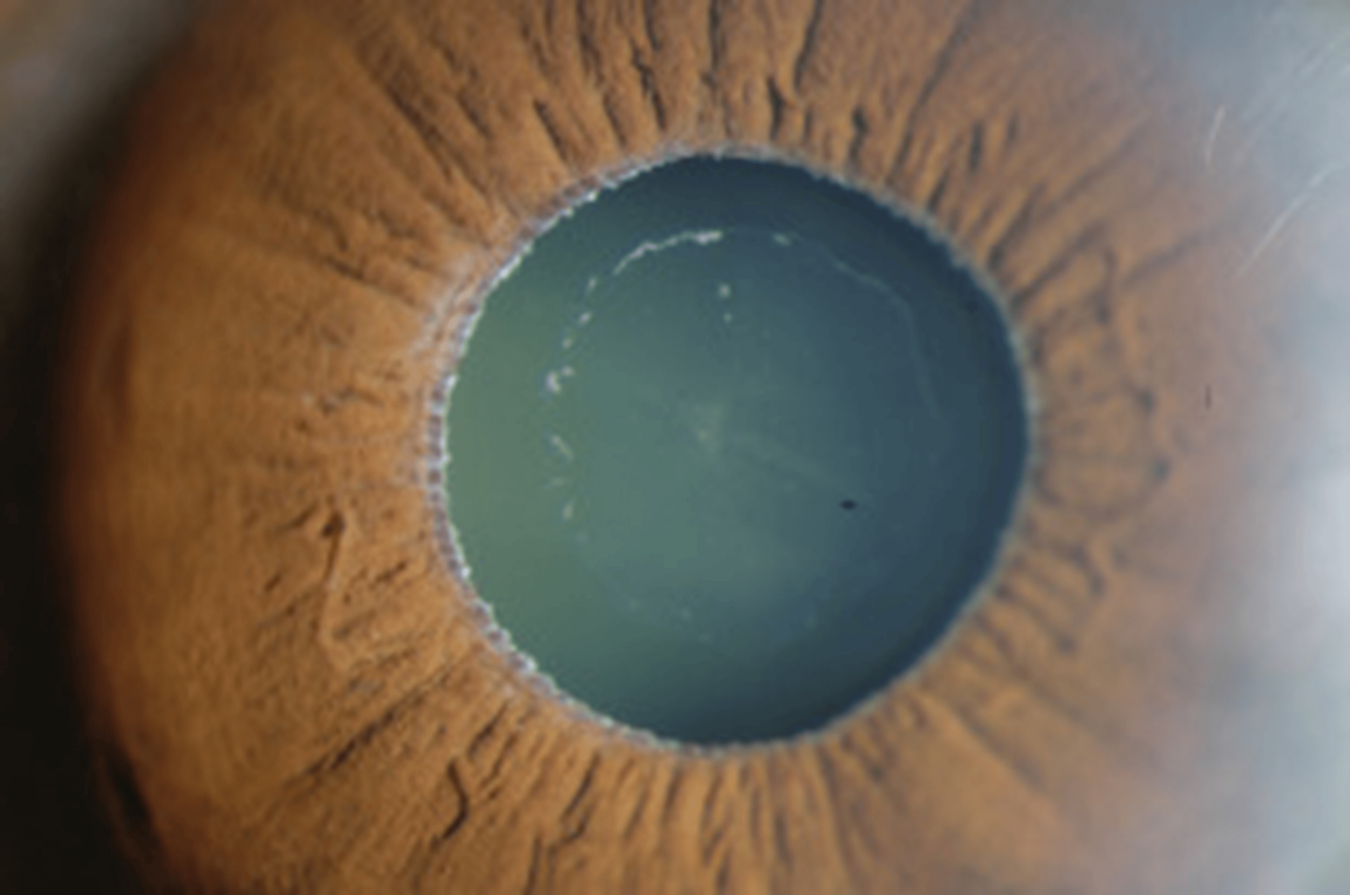
Slit-lamp examination revealed pseudoexfoliative material on the anterior lens capsule.

A 72-year-old woman presented with decreased vision in both eyes, more pronounced in the right eye. Preoperative best-corrected visual acuity (BCVA) in the right eye was approximately 0.1. Slit-lamp examination revealed features consistent with pseudoexfoliation syndrome, including the deposition of pseudoexfoliative material on the anterior lens capsule and poor pupillary dilation (Figure 1).

Cataract surgery was planned for the right eye. Intraoperatively, zonular weakness with areas of zonular dialysis was noted. After successful capsulorhexis, hydrodissection was carefully performed. Due to zonular instability, a “pop-out” nucleus technique was utilized, whereby the nucleus was gently prolapsed into the anterior chamber using controlled hydrodissection while preserving the integrity of the posterior capsule.

The nucleus was then emulsified using phacoemulsification, followed by the removal of the epinucleus and cortical material via irrigation and aspiration. A capsular tension ring (CTR) was implanted to stabilize the capsular bag, and a posterior chamber intraocular lens (IOL) was successfully placed.

On the first postoperative day, visual acuity improved to 0.3 with mild corneal edema. At one-week follow-up, the patient achieved a BCVA of 0.9 with a stable intraocular lens and no complications.

## Discussion

Pseudoexfoliation syndrome (PXF) is a well-recognized age-related systemic condition characterized by the progressive accumulation of fibrillar extracellular material in ocular tissues, particularly on the anterior lens capsule, zonules, and pupillary margin [1]. It is frequently associated with increased surgical complexity during cataract extraction due to zonular weakness, poor pupillary dilation, and a higher risk of intraoperative complications [2].

In the present case, the presence of pseudoexfoliative material on the anterior lens capsule was clearly identified on slit-lamp examination, consistent with classical findings described in the literature [1]. Such cases require careful preoperative planning and intraoperative modifications to ensure surgical safety and optimal outcomes.

Previous studies have consistently reported a higher incidence of intraoperative complications in eyes with pseudoexfoliation syndrome, particularly zonular dialysis, capsular rupture, and vitreous loss [2,4]. Shingleton et al. emphasized that zonular instability remains one of the most significant challenges in managing these cases, often necessitating modified surgical strategies [2].

The pop-out nucleus technique represents an effective alternative approach in managing cataracts associated with pseudoexfoliation. This method minimizes zonular stress by facilitating controlled nucleus prolapse into the anterior chamber, thereby reducing manipulation within the capsular bag. Compared to conventional phacoemulsification techniques, which rely heavily on in-the-bag nucleus disassembly, this approach may decrease mechanical stress on weakened zonules and lower the risk of intraoperative complications [3,5].

Furthermore, Shingleton et al. have highlighted the importance of adapting surgical techniques in eyes with compromised zonular support, recommending strategies that limit zonular traction and enhance intraoperative control [3]. In this context, the pop-out nucleus technique aligns well with these principles and offers a safer surgical alternative in selected cases.

Additionally, the use of adjunctive measures such as capsular tension rings, careful hydrodissection, and viscoelastic support has been shown to improve surgical outcomes in pseudoexfoliation cases [5]. Surgeons should remain vigilant for signs of zonular weakness and be prepared to modify their surgical approach accordingly.

This case underscores the importance of recognizing pseudoexfoliation preoperatively and tailoring the surgical technique to minimize complications. The successful use of the pop-out nucleus technique in this setting demonstrates its value as a practical and effective strategy in managing complex cataract cases associated with pseudoexfoliation syndrome.

## Conclusions

Pseudoexfoliation syndrome remains a significant risk factor for complications during cataract surgery due to zonular instability and poor pupillary dilation. Early recognition through careful slit-lamp examination is essential for appropriate surgical planning. The pop-out nucleus technique offers a safe and effective approach in such cases by minimizing zonular stress and reducing intraoperative risks. This case highlights the importance of adapting surgical strategies to the underlying pathology to achieve optimal outcomes.

## References

[1] Ritch R, Schlötzer-Schrehardt U (2001). Exfoliation syndrome. Surv Ophthalmol.

[2] Shingleton BJ, Crandall AS, Ahmed II (2009). Pseudoexfoliation and the cataract surgeon: preoperative, intraoperative, and postoperative issues related to intraocular pressure, cataract, and intraocular lenses. J Cataract Refract Surg.

[3] Shingleton BJ, Heltzer J, O'Donoghue MW (2003). Outcomes of phacoemulsification in patients with and without pseudoexfoliation syndrome. J Cataract Refract Surg.

[4] Schlötzer-Schrehardt U, Naumann GO (2006). Ocular and systemic pseudoexfoliation syndrome. Am J Ophthalmol.

[5] Belovay GW, Varma DK, Ahmed II (2010). Cataract surgery in pseudoexfoliation syndrome. Curr Opin Ophthalmol.

